# Outcome after primary directed radiotherapy in predominantly PSMA-PET/CT-staged patients with metastatic prostate cancer

**DOI:** 10.1186/s13550-026-01473-1

**Published:** 2026-06-23

**Authors:** Christian Trapp, Sarah Frederike Brose, Michael Keilholz, Minglun Li, Nina-Sophie Schmidt-Hegemann, Claus Belka, Elena Berg, Philipp Weinhold, Jozefina Casuscelli, Christian Stief, Gabriel Sheikh, Paul Rogowski

**Affiliations:** 1 Department of Radiation Oncology, LMU University Hospital, LMU Medizin, Marchioninistr. 15, Munich, 81377 Germany; 2 Department of Radiation Oncology, Klinikum Lüneburg, Lüneburg, Germany; 3 Bavarian Cancer Research Center (BZKF), Munich, Germany; 4 German Cancer Consortium (DKTK), Partner Site Munich, Munich, Germany; 5 Department of Urology, LMU University Hospital, LMU Medizin, Munich, Germany; 6 Department of Nuclear Medicine, LMU University Hospital, LMU Medizin, Munich, Germany

**Keywords:** Prostate cancer, Oligometastatic disease, Radiotherapy, Primary directed therapy

## Abstract

**Background:**

Oligometastatic prostate cancer (OMPC) is typically treated with systemic therapy, which may be complemented by radiotherapy (RT) of the prostate as part of primary directed therapy (PDT). This study retrospectively evaluated clinical outcomes following PDT in OMPC.

**Results:**

Twenty-four consecutive patients who underwent PDT for OMPC between 2011 and 2023 were analyzed retrospectively. The primary endpoint was progression-free survival (PFS); secondary endpoints included biochemical PFS (bPFS), overall survival (OS), and treatment-related toxicity. Survival was estimated using Kaplan–Meier analysis, and prognostic factors for PFS were assessed using univariate log-rank tests and multivariate Cox regression. The median follow-up was 40 months. Median age was 75 years (range: 58–83), with a median of two metastases (range: 1–5). Nineteen patients (79%) were staged with PSMA-PET/CT; 20 had synchronous and 20 low-burden disease (CHAARTED criteria). The most common RT regimen was 60 Gy in 20 fractions. Twenty-three patients received androgen deprivation therapy (ADT), including six with an androgen receptor signaling inhibitor (median duration: 23 months). Additionally, 22 underwent metastasis-directed therapy (MDT), and five elective nodal RT. Median PFS and bPFS were 80 and 73 months, respectively; median OS was not reached. Three-year PFS, bPFS, and OS were 78%, 81%, and 84%. Low metastatic burden (*p* = 0.007) and synchronous metastases (*p* = 0.027) predicted longer PFS. No acute grade 3 toxicity occurred; late grade 3 gastrointestinal and genitourinary toxicity occurred in 8% each.

**Conclusions:**

In this predominantly PSMA-PET/CT-staged cohort, PDT combined with MDT and ADT achieved promising long-term outcomes with acceptable toxicity, warranting confirmation in larger prospective studies.

## Background

Most prostate cancer cases are currently diagnosed at a localized stage. However, in Germany, approximately 18% of patients present with metastatic disease at initial diagnosis [1], corresponding to metastatic hormone-sensitive prostate cancer (mHSPC). For patients with synchronous metastatic disease, the standard treatment approach consists of androgen deprivation therapy (ADT) in combination with an androgen receptor signaling inhibitor (ARSI), such as enzalutamide, apalutamide, darolutamide, or abiraterone. In selected fit patients, the addition of chemotherapy with docetaxel is considered, resulting in a triple therapy regimen [2].

Particularly in patients with oligometastatic prostate cancer (OMPC, mostly defined as 5 or less metastases), some radiation oncology centers have offered additional radiotherapy (RT) of the prostate - referred to as primary-directed therapy (PDT), occasionally combined with metastasis-directed therapy (MDT) targeting metastatic lesions. In recent years, several studies have suggested a significant benefit from adding PDT:

In 2018, the HORRAD trial demonstrated that supplemental PDT improved biochemical progression-free survival (PFS), though not overall survival (OS), in patients with synchronous bone metastases receiving lifelong ADT [3]. The same year, data from the STAMPEDE consortium indicated that PDT improved OS in patients with low metastatic burden (per CHAARTED criteria), but not in those with high metastatic burden [4]. More recently, the 2024 PEACE-1 trial reported that adding PDT improved radiographic PFS in patients with low-volume disease treated with ADT plus docetaxel and abiraterone, but not in patients receiving only ADT plus docetaxel. Furthermore, PDT delayed the onset of castration resistance and reduced genitourinary (GU) toxicities [5]. All three trials relied on conventional imaging for staging. In contrast, randomized evidence now demonstrates the superior accuracy of PSMA-PET/CT compared with conventional imaging [6]. Integration of PSMA-PET/CT into the management of OMPC has the potential to refine patient selection and impact clinical outcomes. However, evidence regarding PSMA-PET/CT–guided PDT remains limited.

The present analysis therefore aims to assess the real-world outcomes and toxicity profiles in OMPC patients, primarily staged with PSMA-PET/CT, who underwent PDT at our center.

## Methods

### Patient population

This analysis includes all OMPC patients who received PDT between November 2011 and September 2023 at the University Hospital of LMU Munich. OMPC was defined as the presence of five or fewer hematogenous metastases. Patients were classified according to the European Society for Radiotherapy and Oncology (ESTRO) and European Organisation for Research and Treatment of Cancer (EORTC) consensus recommendations [7], with all types of oligometastatic disease included.

This retrospective analysis was performed in compliance with the principles of the Declaration of Helsinki and its subsequent amendments [8] and was approved by the local Ethics Committee of the Medical Faculty (approval number 25–0429).

### Radiotherapy treatment and follow-up

Patients receiving normofractionated or moderately hypofractionated RT regimens were treated using conventional linear accelerators employing intensity-modulated radiotherapy (IMRT) or volumetric modulated arc therapy (VMAT) with five fractions per week and daily image-guidance. Patients treated with ultrahypofractionated RT regimens underwent online adaptive magnetic resonance (MR)-guided RT. Concomitant ADT was recommended for all patients. The selection of the specific ADT agent, treatment duration, and the decision to incorporate an additional ARSI were at the discretion of the treating urologist. Follow-up evaluations were conducted initially at three months post-RT and subsequently every six to twelve months.

### Endpoints and statistical analysis

The primary endpoint of the study was PFS. Progression was defined as either biochemical progression (prostate specific antigen [PSA] > post-RT nadir + 2 ng/mL) or radiographic progression. Secondary endpoints included biochemical PFS (bPFS), OS, as well as acute and late toxicity according to Common Terminology Criteria for Adverse Events (CTCAE) v5.0. PFS and bPFS were calculated from the last day of radiotherapy to progression or last follow-up. OS was defined as time from the start of radiotherapy to death from any cause or last follow-up. A sensitivity analysis excluding non-PSMA patients was performed. Statistical analyses were performed using IBM SPSS^®^ version 26.0. Survival outcomes were estimated using the Kaplan–Meier method and compared using the log-rank test. For multivariate analysis, Cox regression analysis was applied to all covariates found to be significant in univariate analysis. Multivariate analyses were considered exploratory due to limited sample size. Reporting of the results followed STROBE guidelines.

## Results

**Fig. 1:**
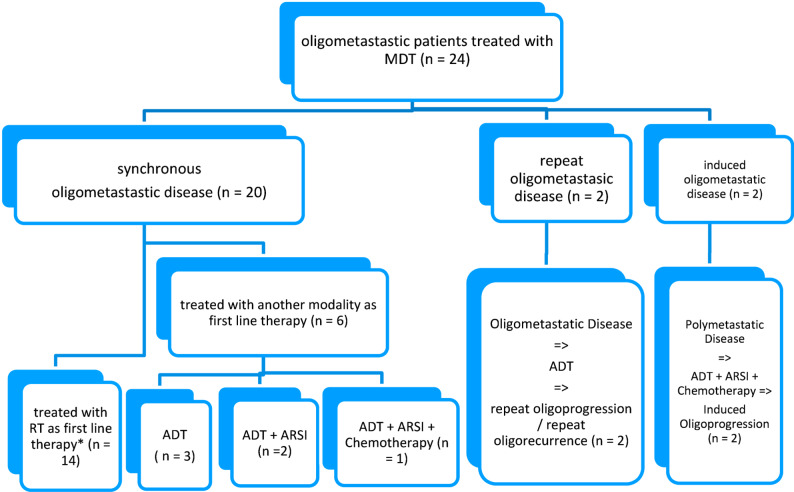
Distribution of Oligometastatic Disease Classes According to the ESTRO/EORTC Framework and Overview of Prior Treatments [7]. *: time from first diagnosis to PDT 0–3 months

A total of 24 patients with OMPC underwent PDT between November 2011 and September 2023. Patient characteristics are summarized in Table 1 and illustrated in Fig. 1.

Twenty patients (83%) presented with synchronous metastases. Of these, 14 patients received PDT as part of first-line treatment, defined as initiation of RT within three months of diagnosis or the start of ADT. The remaining six patients received systemic therapy first - either ADT alone, ADT plus ARSI, or triple therapy - followed by PDT more than three months afterwards. Four patients (17%) had metachronous oligometastatic disease. Among them, two had initially presented with synchronous polymetastatic disease and later developed oligoprogression following triplet therapy. The third patient was initially diagnosed with synchronous oligometastatic disease, responded well to ADT, and experienced a repeat oligorecurrence two years later. The fourth patient initially had synchronous oligometastatic disease and was treated with ADT (including upfront chemotherapy and later enzalutamide), developing repeat oligoprogression three years later. Nearly half of the patients (46%) presented with only a single metastasis. Eighteen patients (75%) had an International Society of Urological Pathology (ISUP) grade group 4 or higher, and eight patients (33%) had concurrent lymph node (LN) metastases. The majority of patients (*n* = 19, 79%) were staged with prostate-specific membrane antigen-based positron emission tomography/computed tomography (PSMA-PET/CT) prior to receiving PDT. In 17 patients (71%), [18 F]PSMA-1007 PET/CT was used, and in two patients (8%), [68Ga]PSMA-11 PET/CT was performed.

**Table 1:** Patient characteristics

Patients, n	24
Age, median (range)	75 (58–83)
Tumor stage, n (%)	
cT1	5 (21%)
cT2	12 (50%)
cT3	5 (21%)
cT4	1 (4%)
Unknown	1 (4%)
Nodal stage, n (%)	
cN0	15 (63%)
cN1	8 (33%)
Unknown	1 (4%)
initial PSA, (ng/ml), median (range)	16,5 (4–1252)
ISUP score, n (%)	
2	1 (4%)
3	5 (21%)
4	9 (38%)
5	9 (38%)
Oligometastatic Disease, n (%)	
Synchronous	20 (83%)
metachronous oligorecurrence	1 (4%)
induced oligoprogression	2 (8%)
repeat oligoprogression	1 (4%)
Imaging modality, n (%)	
PSMA-PET/CT	19 (79%)
[18 F] PSMA-1007 PET/CT	17 (71%)
[68Ga] PSMA-11 PET/CT	2 (8%)
Choline-PET/CT	1 (4%)
Bone scan	4 (17%)
PSA at time of imaging, (ng/ml), median (range)	9,80 (0,02–1252)
ADT at time of imaging	
Yes	12 (50%)
No	12 (50%)
Number of bone metastases at time of PDT*	
1	11 (46%)
2	4 (17%)
3	5 (21%)
4	2 (8%)
5	2 (8%)
Metastatic Burden (according to CHAARTED [9])	
Low	20 (83%)
High	4 (17%)
Time from first diagnosis to PDT	
0–3 months	14 (58%)
> 3 months	10 (42%)
PSA before PDT, (ng/ml), median (range)	9,77 (0,01–1252)

*: in case of metachronous oligorecurrence, induced oligoprogression or repeat oligoprogression number of PET-positive/vital bone metastases

ADT = androgen deprivation therapy; CHAARTED = Chemohormonal Therapy Versus Androgen Ablation Randomized Trial for Extensive Disease in Prostate Cancer [9]; ISUP = International Society of Urological Pathology; n = number; PDT = primary directed therapy; PET/CT = positron emission tomography/computed tomography; PSMA = prostate-specific membrane antigene; PSA = prostate-specific antigen

Treatment details are presented in Tables 2 and 3. The most frequently applied fractionation schemes for PDT were 60 Gy in 20 fractions (8 patients, 33%), followed by 70 Gy in 28 fractions (25%), and 74 Gy in 37 fractions (4 patients, 17%). The median RT dose, converted to an equivalent dose in 2 Gy fractions using an α/β ratio of 1.5 Gy (EQD2_α/β=1.5_) was 77.1 Gy (range 50 to 90.6 Gy). Additionally, 5 patients (21%) received elective nodal RT (ENRT) to the pelvic lymphatic pathways, mostly due to pelvic lymph node metastases. A total of 22 patients (92%) additionally underwent MDT, encompassing treatment of 40 metastatic lesions in total. Nineteen patients (79%) were treated at all lesions. In cases where not all lesions were treated, the decision was based on lesion characteristics such as very small size, absence of a CT-morphologic correlate, or PET findings indicating non-viable (avital) metastases. The median EQD2_α/β=1.5_ for MDT was 62.9 Gy (range 64.3 to 77.1 Gy). Metastatic sites located in close anatomical proximity to the prostate or to the ENRT target volume were included in the respective treatment fields and consequently received a normofractionated or moderately hypofractionated schedule. Distant metastatic lesions were predominantly managed with stereotactic body radiotherapy (SBRT), most frequently delivered as 40 Gy in 10 fractions or 30 Gy in 5 fractions, prescribed to the 80% isodose line. Twenty-three patients (96%) underwent concurrent ADT, with 19 of them receiving it for more than one year. Additionally, 6 patients (25%) were treated with an ARSI.

**Table 2:** Treatment characteristics

ADT	
Concomitant ADT	
yes	23 (96%)
no	1 (4%)
Additional ARSI	
no	18 (75%)
Abiraterone	3 (13%)
Enzalutamide	2 (8%)
Darolutamide	1 (4%)
ADT still ongoing at end of FU	
yes	10 (42%)
no	14 (58%)
Duration of ADT (only patients without ADT at end of FU, *n* = 14)	
6–11 months	4 (31%)
12–23 months	4 (31%)
24 or more months	5 (38%)
unknown	1 (4%)
Duration of ADT (only patients with ongoing ADT at end of FU, *n* = 10)	
6–11 months	0
12–23 months	4 (40%)
24 or more months	6 (60%)
Neoadjuvant ADT*	
yes	13 (54%)
no	11 (46%)
Duration of neoadjuvant ADT in months, median (range)	4 (1–42)
**MDT**	
Additional MDT	
yes	22 (92%)
no	2 (8%)
All metastases treated	
yes	19 (79%)
no	5 (21%)
**ENRT**	
Additional ENRT	
yes	5 (21%)
no	19 (79%)

* started more than one month before start of PDT

ADT = androgen deprivation therapy; ARSI = androgen receptor signaling inhibitor; ENRT = elective nodal radiotherapy; FU = follow up; MDT = metastasis directed therapy; PDT = primary directed therapy

**Table 3:** Fractionation schemes for PDT

Number of fractions	Dose per fraction (Gy)	Total dose (Gy)	BED_α/β=1.5_ (Gy)	EQD2_α/β=1.5_ (Gy)	Number of patients
5	7,25	36,25	211,50	90,60	1 (4%)
7	6,00	42,00	210,00	90,00	1 (4%)
28	2,50	70,00	186,70	80,00	6 (25%)
20	3,00	60,00	180,00	77,10	8 (33%)
26	2,50	65,00	173,30	74,30	1 (4%)
37	2,00	74,00	172,70	74,00	4 (17%)
35	2,00	70,00	163,30	70,00	1 (4%)
20	2,75	55,00	155,80	66,80	1 (4%)
25	2,00	50,00	116,70	50,00	1 (4%)

### Oncologic outcome

Median follow-up was 40 months. Of the 23 patients who received concomitant ADT, 10 remained on ADT at the end of the follow-up period.

Biochemical progression occurred in seven patients. All seven of these patients, along with one additional patient, demonstrated clinical disease progression on restaging imaging. New osseous metastases were observed in 7 patients (29%), and 4 of them (17%) underwent repeat MDT for the new lesions. Among these 7 patients, 2 (8%) also experienced local recurrences after receiving EQD2_α/β=1.5_ doses of 50.0 Gy and 77.1 Gy, respectively. Additionally, 3 of these 7 patients and 1 other patient (a total of 4 patients, 17%) developed LN metastases. Notably, none of these 4 patients had received prior ENRT.

**Fig. 2:**
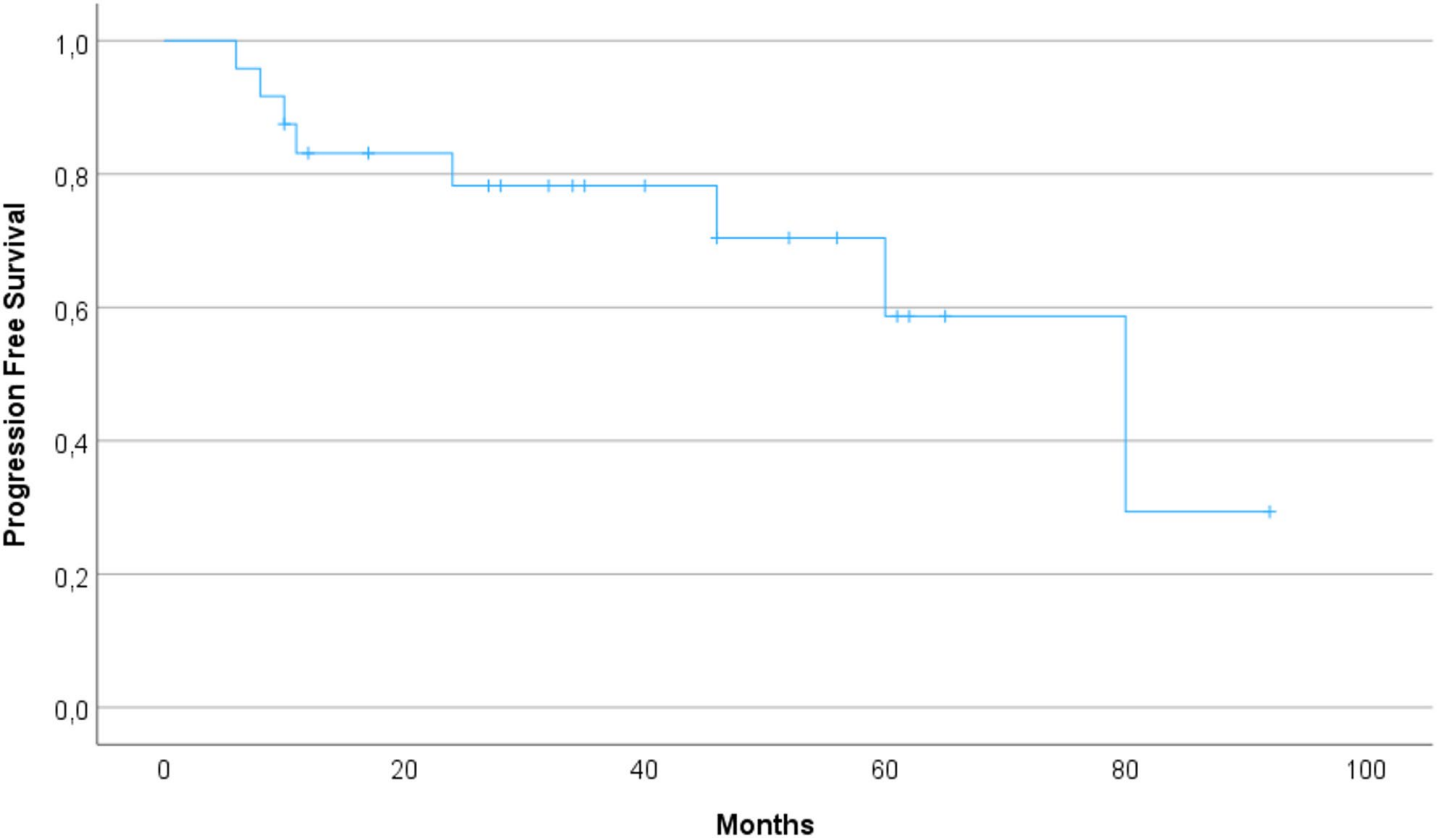
Progression Free Survival

The median PFS was 80 months, with a 3-yr PFS of 78% (see Fig. 2). Among patients who were no longer on ADT at the final follow-up, the 3-yr PFS was also 78%. Median bPFS was 73 months, with a 3-yr bPFS of 81%. Among patients who were no longer on ADT at the final follow-up, the 3-yr bPFS was also 77%.

**Fig. 3:**
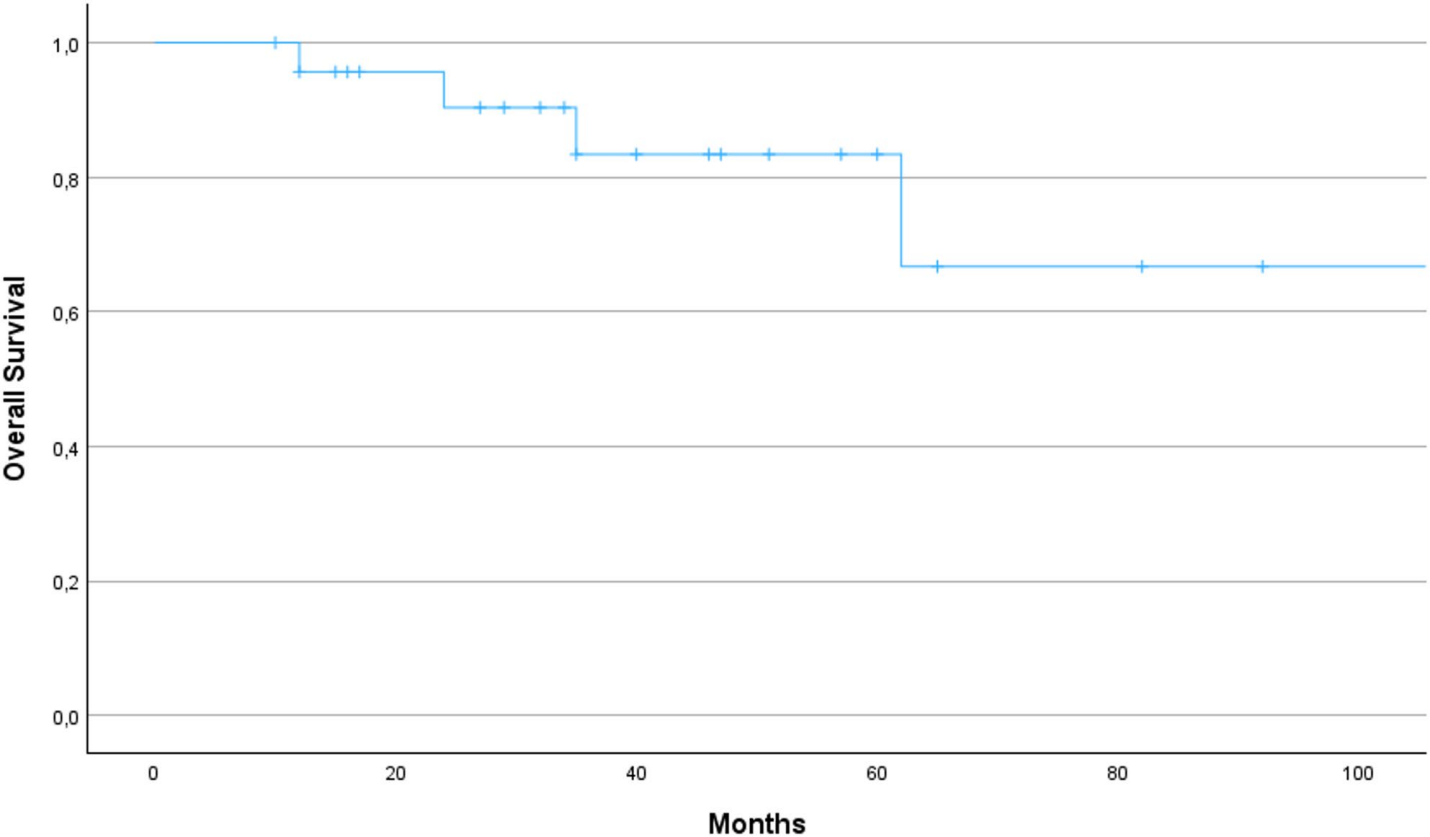
Overall survival

In total, four patients died during the follow-up period, with only one death attributed to prostate cancer. The median OS was not reached, and the 3-yr OS was 84% (see Fig. 3). Among patients who were no longer receiving ADT at the last follow-up, 3-yr OS was 85%.

In the sensitivity analysis excluding patients who had not undergone staging with PSMA PET/CT (*n* = 5; 21%), the median PFS, bPFS, and OS were 45, 47, and 53 months, respectively. The 3-year PFS, bPFS, and OS rates were 72%, 75%, and 76%, respectively.

Table 4 demonstrates the results of the uni- and multivariate analyses. A low metastatic burden (according to CHAARTED [9]) and synchronous OMPC were significantly associated with improved PFS in the multivariate analysis.

**Table 4:** Uni- and multivariate analysis for factors associated with PFS

Patient characteristics	*n*		univariate		multivariate	
			median PFS	*p*-value	HR (95% CI)	*p*-value
**ISUP Score**				0.094		
≤ 3	5		80			
≥ 4	19		60			
**Initial tumor stage**				0.452		
≤ T2	17		80			
≥ T3	6		NR			
**Initial nodal stage**				0.596		
N0	15		NR			
N1	8		80			
**Metastatic Burden**				**< 0.001**	0.03 (0.00–0.39)	**0.007**
Low	20		80			
High	4		8			
**Initial PSA**				0.087		
< 20 ng/ml	13		NR			
≥ 20 ng/ml	11		60			
**Imaging modality**				0.245		
PSMA-PET/CT	19		45			
Other	5		79			
**Dose (EQD2**_**α/β=1.5**_ **)**				0.471		
< 77,1 Gy	8		80			
≥ 77, 1 Gy	16		NR			
**ADT duration**				0.577		
< 12 months	5		NR			
≥ 12 months	19		80			
**Additional ARSI**				**0.024**	3.05 (0.61–15.18)	0.173
yes	6		11			
no	18		80			
**All metastases treated by MDT**				0.298		
yes	19		NR			
no	5		80			
**Oligometastatic disease**				**0.008**	0.12 (0.02–0.78)	**0.027**
synchronous	20		80			
all others	4		10			

ADT = androgen deprivation therapy; CI = confidence interval; HR = hazard ratio; ISUP: International Society of Urological Pathology; NR = not reached; PSA = prostate-specific antigen; sRT = salvage radiotherapy

### Toxicity

In this analysis we observed no acute toxicity ≥ °3 at all. Two patients (8%) developed late ≥ °3 gastrointestinal (GI) toxicity. Both patients required endoscopic interventions due to radiation-induced proctitis with recurrent rectal bleeding events. Two patients (8%) developed late ≥ °3 genitourinary (GU) toxicity. One patient developed a radiation-induced cystitis with macrohematuria and required cystoscopic interventions, the other patient underwent a transurethral resection of the prostate (TUR-P) due to obstructive symptoms.

## Discussion

In the present analysis, patients with OMPC treated with PDT achieved a 3-year PFS rate of 78%, and a 3-year OS rate of 84%. In a sensitivity analysis excluding patients who were not staged with PSMA-PET/CT 3-year PFS and OS were 72% and 76%. Given the overall small sample size, we believe that these variations are most likely attributable to statistical fluctuation rather than a true effect of staging modality. Furthermore, imaging modality was not a significant factor in the univariate analysis. No acute grade ≥ 3 toxicities were observed, whereas late grade ≥ 3 GI and GU toxicities occurred in 8% and 8% of patients, respectively. On multivariate analysis, low metastatic burden and synchronous OMPC emerged as independent predictors of improved PFS.

These outcomes are consistent with the available evidence from prospective trials and retrospective series (Table 5):

Among randomized trials, HORRAD enrolled 432 patients with synchronous mHSPC randomized to ADT ± PDT. No OS benefit was observed (median 45 vs. 43 months), although biochemical PFS improved with PDT [3]. The negative OS result is generally attributed to the unfavorable patient profile, with > 5 metastases in most patients and baseline PSA > 100 ng/ml.

The STAMPEDE consortium conducted a randomized, prospective trial including more than 2,000 patients with conventionally staged, newly diagnosed mHSPC and tested PDT in addition to ADT ± chemotherapy [4]. While overall OS was unchanged, a clear OS benefit emerged in the subgroup with low metastatic burden according to CHAARTED criteria (median OS 86 vs. 64 months; 3-year OS 81% vs. 73%). These results closely resemble those of the present cohort (3-year OS 84%). Of note, 18% of patients in both treatment arms also received chemotherapy. The STOPCAP meta-analysis, pooling HORRAD and STAMPEDE, confirmed the benefit of PDT in patients with < 5 metastases (HR 0.73), while demonstrating no advantage in unselected populations [10].

**Table 5:** Overview of evidence for PDT

Author	Year	Study type	*n* (patients)	Maximal number of metastases	Patient characteristics	M1b-disease	Treatment	Median dose PDT	Systemic therapy	Staging with PSMA-PET/CT	Overall survival	Biochemical progression-free survival	Clinical progression-free survival	Toxicity
**Riva **[11]	2017	retrospective cohort study	20	≤ 5 bone metastases	synchronous mHSPC	1	PDT + palliative RT of bone metastases	median BED 173 Gy	ADT 100%, no ARSI/CTx reported	0%, (Cholin-PET/CT used, proportion n.r.)	2-yr: 100%	biochemical failure: 12/20 patients after median 23 months	clinical progression: 12/20 patients after median 24 months	acute G3: 5% late G3: 0%
**Boeve (HORRAD) **[3]	2019/2021	randomized controlled trial	432	no limitation, median 5–15	synchronous mHSPC	1	ADT vs. ADT + PDT	70 Gy/35 fx 57.76/19 fx	ADT 100%	0%	median 43 vs. 45 months	median 12 vs. 15 months	n.r.	temporary modest urinary and bowel symptoms after ADT + RT
**Parker (STAMPEDE) **[4]	2018/2022	randomised controlled phase 3 trial	2061	no limitation, low metastatic burden: 40%	synchronous mHSPC	89%%	ADT vs. ADT + PDT	55 Gy/20 fx 36 Gy/6 weekly fx	ADT 100% + Docetaxel in 18%	0%	all patients: 3-yr: 62% vs. 65% low metastatic burden: 3-yr: 73% vs. 81% 5-yr: 53% vs. 65%	n.r.	all patients: 3-yr PFS: 44% vs. 44% low metastatic burden: 3-yr PFS: 58% vs. 63%	radiotherapy-related: acute ≥ G3: 6% late: ≥ G3: 6%
**Deantoni **[12]	2020	retrospective cohort study	39	≤ 2	synchronous (54%) and metachronous OMPC	1	PDT + MDT	EQD2 88 Gy	ADT (100%)	0%	4-yr: 82%	4-yr: 53%	4-yr cPFS: 66% 4-yr FFDM: 73%	≥G3: 5%
**Reverberi **[13]	2020	retrospective cohort study	37	≤ 5	synchronous OMPC	0,7	PDT + MDT	68.75 Gy in 25 fx	ADT 97%, + Docetaxel in 3%	0% (100% Cholin-PET/CT)	2-yr: 97% 5-yr: 65%	2-yr: 73% 5-yr: 35%	2-yr rPFS: 79% 5-yr rPFS: 55%	no ≥G3
**Imber **[14]	2020	retrospective cohort study	47	≤ 6	synchronous OMPC	0,92	PDT + MDT	various, normo-fractionated 75–81 Gy most common	ADT 100% + Abiraterone in 26%	9%	2-yr: 87%	2-yr: 77%	2-yr: DMFS: 68%	n.r.
**Morgan **[15]	2021	retrospective cohort study	282 ADT/ 128 ADT + PDT	n.r.	synchronous mHSPC	0,85	PDT (+ MDT in 4%)	EQD2 ≥ 47,6 Gy	ADT 100%, no ARSI/CTx	n.r.	2-yr: 53% vs. 75% with RT 5-yr: 25% vs. 41% with RT	n.r.	n.r.	n.r.
**Montero **[16]	2021	retrospective cohort study	26	n.r.	synchronous mHSPC	0,3	PDT + MDT	63 Gy in 21 fx	ADT 100%, + Abiraterone or Docetaxel in 19%	0%, (Cholin-PET/CT used, proportion n.r.)	crude OS 100% after mFU of 16 months	n.r.	2-yr PFS: 85%	no ≥G3
**Inaba **[17]	2021	retrospective cohort study	35	n.r., low metastatic burden: 94%	synchronous mHSPC	1	PDT + MDT	72 Gy in 36 fx	ADT 100%, no ARSI/CTx reported	n.r.	3-yr: 94% 8-yr: 81%	3-yr: 68% 8-yr: 57%	3-yr cPFS: 88% 8-yr cPFS: 53%	acute G3: 9%; late G3: 3%
**Bossi (PEACE-1) **[5]	2024	randomised, controlled, phase 3 trial	1172	no limitation, low metastatic burden: 43%	synchronous mHSPC	n.r.	ADT vs. ADT + PDT vs. ADT + Abiraterone vs. ADT + Abiraterone + PDT	74 Gy in 37 fx	ADT (100%) + Docetaxel in 61% +	0%	median 6.9 yrs without PDT vs. 7.5. yrs with PDT	n.r.	median rPFS 3.1 yrs (ADT + Abiraterone) vs. 7.5 yrs (ADT + Abiraterone + PDT)	radiotherapy-related ≥G3: GU ≤ 1.9%; GI: ≤1.5%;
**Poon **[18]	2025	prospective observational study	43	≤ 5 pelvic lymph node or distant metastases (M1: 72%)	synchronous OMPC	n.r.	PDT + MDT	36.25–40 Gy in 5 fx, 42 Gy to DIL	ADT 100% + ARSI in 52%	100%	3-yr: 100%	3-yr: 93%	3-yr PFS: 93%	no radiotherapy-related ≥G3
**Trapp ** **(current study)**	2025	retrospective cohort study	24	≤ 5	synchronous (83%) and metachronous OMPC	1	PDT + MDT	60 Gy/20 fx	ADT (96%) + ARSI in 25%	79%	2-yr: 91% 3-yr:84% 4-yr: 84% 5-yr: 84%	3 year: 81%	median PFS: 6.7 yrs 2-yr PFS: 78% 3-yr PFS: 78% 4-yr PFS: 70%	acute G3: 0%; late G3 GU: 8% late G3 GI: 8%

ADT: androgen deprivation therapy; ARSI: androgen receptor signalling inhibitor; BED: biologically effective dose; bPFS: biochemical progression-free survival; cPFS: clinical progression-free survival; CTx: chemotherapy; DMFS: distant metastasis-free survival; EQD2: Equivalent Dose in 2 Gy Fractions; FFDM: freedom from distant metastasis; fx: fractions; G3: grade 3; Gy: gray; MDT: metastasis-directed therapy; mHSPC: metastatatic hormone-sensitive prostate cancer; n: number; n.r.: not reported; OMPC: oligometastatic prostate cancer; OS: overall survival; PDT: primary-directed therapy; PFS: progression-free survival; PSMA-PET/CT: prostate-specific membrane antigene-positron emission tomography/computed tomography; rPFS: radiological progression-free survival; RT: radiotherapy; yr: year

In 2024, the randomized, prospective PEACE-1 trial evaluated the role of PDT in conventionally staged patients with synchronous mHSPC. Among patients with low-volume disease, PDT significantly prolonged radiographic PFS when administered in combination with standard of care (ADT ± chemotherapy) plus abiraterone (median 7.5 vs. 4.4 years), but not in the absence of abiraterone. However, no OS improvement was seen overall, and half of the patients also received chemotherapy [5]. Notably, 50% of patients with low-volume disease in PEACE-1 also received chemotherapy. By contrast, in the present analysis only 25% of patients received an ARSI, yet the median radiographic PFS was 6.7 years.

Based on this prospective evidence, PDT has been incorporated in international clinical guidelines for synchronous, low-volume mHSPC [2, 11]. However, patients enrolled in the mentioned prospective trials were staged using conventional imaging, limiting their transferability to today’s PSMA-PET/CT era. In addition, patients in these trials did not receive additional MDT.

Apart from the mentioned prospective trials there are several single-center retrospective analyses reporting on the outcomes of PDT.

Riva et al. reported the outcomes of 20 patients with de novo synchronous metastatic disease and ≤ 5 bone metastases. PDT + palliative radiotherapy of bone metastases yielded a 2-yr OS of 100%. However, 60% of patients progressed after a median of 24 months [12].

Morgan et al. analysed 410 newly diagnosed metastasized PC patients treated with ADT, of whom 128 also received a PDT (≥ 40 Gy in 15 fractions). PDT was associated with an OS benefit (median 47 vs. 26 months) and a significant dose–response relationship was also observed [13]. However, the median OS was markedly lower than in other trials and in the present analysis. Importantly, no data on metastatic burden or imaging modality were provided.

Moreover, several retrospective analyses have evaluated the combination of ADT, MDT, and PDT, allowing for a meaningful comparison with the present study, in which 92% of patients also received additional MDT.

Deantoni et al. analysed 39 conventionally staged PC patients with one or two synchronous bone metastases treated with PDT, MDT, and ADT [14]. Reported outcomes (4-yr OS 82% and 4-yr metastasis free survival (MFS) 73%) closely mirror those of the present study (4-yr OS 84%, 4-yr PFS 70%).

Reverberi et al. reported on 37 Choline-PET/CT-staged patients with de novo OMPC. The reported outcomes included a 2-yr and 5-yr OS of 97% and 65%, respectively, and a 2-yr and 5-yr biochemical recurrence free survival (BRFS) of 73% and 39% [15]. These results appear somewhat lower to those of the present analysis (2-yr OS 91%, 5-yr OS 84%; 2-yr PFS 78%, 5-yr PFS 59%).

Imber et al. analysed 47 patients with 1–6 synchronous metastases (mainly osseous), 4 of whom (9%) underwent PSMA-PET/CT staging. Systemic treatment included ARSI or chemotherapy in 32%. PDT was heterogeneous, comprising SBRT, conventionally fractionated RT, and brachytherapy; ENRT was applied in two-thirds of patients. Reported outcomes (2-yr OS 87%, 2-yr DMFS 69%) were comparable to our results (2-yr OS 91%, 2-yr PFS 78%) [16].

Montero et al. analysed 26 patients with synchronous osseous (31%) or nodal metastases. All received ADT, with systemic intensification in five cases. PDT was delivered at 63 Gy in 21 fractions. Outcomes (1-yr PFS 94%, 2-yr PFS 85%) are difficult to compare due to the small sample size and limited number of patients with osseous disease [17].

Inaba et al. evaluated 35 PC patients with bone metastases treated with ADT, MDT, PDT and ENRT (regardless of LN involvement). 3-yr OS and PFS were 94% and 88%, respectively, which appear to exceed the corresponding results of the present analysis (84% and 78%, respectively). Notably, no information was provided regarding the use of ARSIs, chemotherapy, or the imaging modalities employed [18].

Most recently, Poon et al. reported on 43 PSMA-PET/CT-staged patients with synchronous OMPC treated with MR-guided SBRT. OMPC was defined as ≤ 5 pelvic nodal or distant metastases; however, only 71% were truly metastatic (M1), while 29% had N1-only disease. Moreover, the proportion of patients with osseous metastases – a key prognostic factor - was not specified. Patients received simultaneous SBRT to the prostate (33.5–40 Gy in 5 fractions) and to all metastatic sites. More than half of the patients also received an ARSI. The 3-yr OS and PFS rates were 100% and 95%, respectively, markedly exceeding those observed in the present analysis (84% and 78%, respectively), with no grade ≥ 3 radiotherapy related toxicity reported [19]. However, these highly favourable results are difficult to compare directly, given the unknown proportion of patients with bone metastases. In summary, studies combining ADT, PDT, and MDT have consistently shown highly encouraging results.

A critical consideration when interpreting our results is the potential for stage migration due to the use of PSMA-PET/CT (Will Rogers phenomenon). Compared with conventional imaging, PSMA-PET/CT detects metastatic disease at lower tumor burden and may reclassify patients who would previously have been considered non-metastatic. This results in a selection of patients with more indolent tumor biology and may partially explain favorable outcomes.

However, despite this potential selection advantage, survival outcomes in the present study were comparable rather than clearly superior to those reported in historical trials based on conventional imaging. This may be explained by the small sample size, treatment heterogeneity, and inclusion of biologically less favorable subgroups, such as patients with oligoprogressive disease.

While our results appear favorable when compared with the three major randomized trials based on conventional staging, such cross-trial comparisons must be interpreted with caution.

With respect to MDT, evidence from several prospective trials is available [20–22]; The ORIOLE trial is particularly noteworthy, as a post-hoc analysis demonstrated that treatment of all PSMA-PET/CT–avid lesions was associated with prolonged distant metastasis-free survival (DMFS). However, high-level evidence specifically addressing synchronous metastatic prostate cancer is still lacking. Therefore, the current EAU guidelines recommend offering MDT only in clinical trials or cohort studies [11].

Despite encouraging outcomes, treatment-related toxicity and quality of life remain essential considerations in this palliative metastatic setting. In the present analysis, no acute grade ≥ 3 toxicities were observed. Late grade ≥ 3 GI and GU toxicities occurred in two patients (8%) and two patients (8%), respectively. These rates are comparable to those reported in the literature (STAMPEDE trial: approximately 6% acute and 6% late toxicity) [4], but slightly higher than the remarkably low toxicity rates observed in the prospective PEACE-1 trial (≤ 2% for both GU and GI) [5].

Notably, PDT may not only be well tolerated but could also help prevent GU complications. In PEACE-1, PDT was associated with fewer adverse events (12.2% vs. 22.3%) and longer survival free from severe GU complications. This effect was consistently observed across all subgroups, supporting consideration of PDT in all patients with newly diagnosed mHSPC [5].

The positive predictors identified in the present analysis (low metastatic burden and synchronous metastatic disease) are consistent with findings from previous studies [4, 10]. Likewise, the observation that patients with de novo OMPC achieve more favourable oncologic outcomes compared with those who are initially polymetastatic or repeatedly oligoprogressive is not unexpected. However, these findings must be interpreted with particular caution given the very small subgroup sizes in the present study and should be considered exploratory.

An unresolved issue regarding PDT is the management of initially polymetastatic patients who respond well to systemic therapy and subsequently convert to an induced oligometastatic state. Overall, the impact of PDT on oncologic outcomes will need to be continuously reassessed in the context of the rapidly evolving landscape of systemic therapies. Nonetheless, based on the PEACE-1 trial, the use of PDT can be justified purely from a symptomatic perspective [5]. Some important insights in the context of multimodal treatment are expected from the STAMPEDE2 trial, although results are not anticipated until 2032. This trial will randomize patients with OMPC in two comparisons: first, between standard of care (ADT + ARSI + chemotherapy + PDT) versus standard of care plus MDT; and second, between standard of care and standard of care plus Lutetium-PSMA therapy (NCT06320067).

This study has several important limitations. First, the small sample size limits statistical power and precludes definitive conclusions; all multivariate analyses should therefore be considered exploratory. Second, the retrospective design introduces potential selection bias and treatment heterogeneity. Third, although the majority of patients (79%) were staged using PSMA-PET/CT, a subset underwent conventional imaging, introducing additional heterogeneity. Fourth, the inclusion of patients with metachronous and oligoprogressive disease - entities with distinct biological behavior - may confound outcome interpretation. Finally, stage migration due to PSMA-PET/CT (Will Rogers phenomenon) likely contributed to patient selection and must be considered when comparing results with historical cohorts.

## Conclusions

The present study evaluates the clinical outcomes of PDT in patients with OMPC. The vast majority of patients were staged using PSMA-PET/CT, and nearly all received concomitant ADT and MDT. Additionally, one-quarter of the patients were treated with an ARSI. The oncologic outcomes in this predominantly PSMA-PET/CT–staged cohort are encouraging but should be interpreted with caution given the small sample size and cohort heterogeneity. Larger series and prospective studies are needed to establish standardized treatment protocols and to address outstanding questions regarding optimal RT doses and integration with systemic therapy.

## Data Availability

Research data are stored in an institutional repository and will be shared upon request to the corresponding author.

## Abbreviations

ADT: Androgen deprivation therapy
ARSI: Androgen receptor signaling inhibitor
BRFS: Biochemical recurrence-free survival
CI: Confidence interval
EAU: European association of urology
EQD2_α/β=1.5_: Equivalent dose in 2 Gy fractions using an α/β ratio of 1.5 Gy
ENRT: Elective nodal radiotherapy
GU: Genitourinary
HR: Hazard ratio
ISUP: International society of urological pathology
LN: Lymph node
MDT: Metastasis directed therapy
MFS: Metastasis-free survival
mHSPC: Metastatic hormone sensitive prostate cancer
MR: Magnetic resonance
NCCN: National comprehensive cancer network
NR: Not reached
OMPC: Oligometastatic prostate cancer
OS: Overall survival
PC: Prostate cancer
PDT: Primary directed therapy
PET/CT: Positron emission tomography/computed tomography
PFS: Progression-free survival
PSA: Prostate specific antigen
PSMA: Prostate specific membrane antigen
RT: Radiotherapy
SBRT: Stereotactic body radiotherapy
TUR-P: Transurethral resection of the prostate

## Declarations

### Statement on the use of generative AI and AI-assisted technologies in the writing process

Generative AI and AI-assisted technologies were used in the preparation of this manuscript to support language refinement, text structuring, and translation. The authors take full responsibility for the content, have critically reviewed all text, and approved the final version of the manuscript.
